# Serum Inflammatory and Prooxidant Marker Levels in Different Periodontal Disease Stages

**DOI:** 10.3390/healthcare9081070

**Published:** 2021-08-20

**Authors:** Ricardo Andreu, Sergio Santos-del-Riego, Francisco Payri

**Affiliations:** 1 Investigation Unit in Integration and Health Promotion (INTEGRA SAÚDE), Department of Physiotherapy, Medicine and Biomedical Sciences, Faculty of Health Sciencies, University of A Coruña, 15071 A Coruña, Spain; sergio.santos.delriego@udc.es; 2 CMT, Polytechnic University of Valencia, Camí de Vera, 46022 Valencia, Spain; fpayri@mot.upv.es

**Keywords:** periodontal diseases, periodontitis, inflammation, 8-hydroxy-2′-deoxyguanosine, malondialdehyde, oxidative stress

## Abstract

Background: Periodontitis has been associated to systemic diseases and this association could be due to an increase in circulating inflammatory and oxidative stress biomarkers in the periodontal disease. This study aimed to evaluate the relationship between inflammatory and pro-oxidant markers according to different stages of periodontitis. Methods: This cross-sectional study included 70 subjects who were divided into three groups according to periodontitis stage: stage II (n = 22), stage III (n = 30), and stage IV (n = 18). We evaluated periodontal parameters and levels of high-sensitivity C-reactive protein (hsCRP), fibrinogen, and malondialdehyde (MDA) in serum, and 8-hydroxy-2′-deoxyguanosine (8-OHdG) in urine. Results: Serum hsCRP and fibrinogen levels were associated with periodontitis severity, which were higher in stage IV than in stages III and II of periodontitis (*p* = 0.003 and *p* = 0.025, respectively). We observed a slight yet insignificant increase in MDA levels related to periodontitis severity. Probing depth and clinical attachment loss were associated with serum fibrinogen and hsCRP levels. However, there were no significant associations between periodontal variables and MDA and 8-OHdG levels. Conclusion: Our data support an association between periodontitis and systemic inflammation, which increases with periodontal disease severity. This indicates the importance of the early diagnosis and treatment of periodontal disease to avoid the development or worsening of systemic inflammatory diseases.

## 1. Introduction

Periodontitis is a very prevalent chronic disease that ranks sixth among the most common pathologies that affect humans [1]. Recently, many publications have reported that periodontitis is related to various systemic diseases, such as cardiovascular diseases [2]. Various systemic pathologies are linked to periodontitis through low-grade chronic inflammation [3,4].

Acute phase reactants, such as high sensitivity C-reactive protein (hsCRP) and fibrinogen, are proteins whose plasma concentrations increase or decrease by at least 25% during inflammatory processes [5]. These proteins are produced by the liver following the stimulation of proinflammatory cytokines synthesised by endothelial cells. hsCRP levels are used to determine serum CRP concentrations lower than 1 mg/dL to discriminate small increases due to subclinical inflammatory processes [6]. Moreover, hsCRP is used to assess cardiovascular risk [7]. Fibrinogen can increase between 2 and 20 times if there are inflammatory processes [6].

Polymorphonuclear neutrophils, fundamental innate immune response cells, generate proinflammatory cytokines, metalloproteinases, and reactive oxygen species (ROS), which are crucial in the development and progression of periodontal damage [8]. Accordingly, malondialdehyde (MDA) is an organic compound resulting from the oxidation of polyunsaturated fatty acids in cell membranes. MDA is generated when a hydroxide radical interacts with lipids, captures a hydrogen atom from the methylene carbon, and causes a very unstable lipid radical, which consequently reacts with another lipid and causes a chain reaction leading to the formation of highly toxic aldehydes [9,10]. In the cell nucleus and mitochondria, oxidative damage to DNA produces 8-hydroxy-2′-deoxyguanosine (8-OHdG), which is a predominant form of deoxyguanosine resulting from guanine oxidation. 8-OHdG urine quantification estimates DNA damage caused by both endogenous and exogenous agents [11].

Over the past few years, numerous clinical and basic experimental studies have shown a strong association between oxidative stress and periodontitis, but most of these periodontal studies evaluated total antioxidant capacity, total oxidant status, and oxidative stress index [12]. However, other recent studies evaluated oxidative stress markers, such as MDA and 8-OHdG levels, but mainly at the local level in saliva [13,14,15,16] and gingival crevicular fluid [14,17] and only a few studies evaluated MDA and 8-OHdG levels in serum [13,18,19]. Although these studies have different results, associations between periodontitis and MDA and 8-OHdG levels have been observed [18,19,20]. Thus, the association between periodontitis and circulating oxidative stress biomarkers could indicate that periodontitis could contribute to the development and progression of several systemic inflammatory diseases [21], whether mitochondrial dysfunction is triggered due to an exacerbated inflammatory process [22]. Therefore, this study aimed to evaluate the relationship between inflammatory and pro-oxidant markers according to different periodontitis stages to determine whether an advanced stage of periodontitis could affect the systemic health of patients.

## 2. Materials and Methods

### 2.1. Study Population

Study participants aged between 30 and 75 years were consecutively recruited at our private dental clinic (Paterna, Valencia, Spain) between June 2019 and March 2020 for this cross-sectional study. Participants were diagnosed with periodontitis when an interdental clinical attachment loss (CAL) in ≥2 non-adjacent teeth or a buccal or oral CAL ≥3 mm with pocketing >3 mm in ≥2 teeth is detected according to the 2017 World Workshop definition [23]. Exclusion criteria were fewer than 14 teeth, infectious or other oral inflammatory diseases, comorbidities such as diabetes or obesity, receipt of periodontal treatment in the last 6 months or antibiotics in the previous 3 months, being under systemic anti-inflammatory treatment, pregnancy, lactation, severe disease including malignancies, alcohol, or drug abuse. Data on smoking were recorded.

This human observational study was conducted according to Strengthening the Reporting of Observational Studies in Epidemiology (STROBE) guidelines and is in accordance with the ethical principles stated in the Declaration of Helsinki. All procedures were approved by the ethics committee of the Arnau de Vilanova-Lliria (Valencia, Spain) hospital (protocol RAM-LIG-2019-01), and all participants gave written informed consent.

### 2.2. Clinical Periodontal Determinations

Periodontal examinations were conducted by an experienced dentist (R. Andreu). Periodontal assessments included measurements of probing depth (PD), CAL, number of sites with PD ≥4 mm, percentage of sites with PD 1–3 mm, 4–5 mm, and ≥6 mm, gingival bleeding on probing (BOP), and plaque index, which were recorded using a manual periodontal probe PCP UNC-15 (Hu-Friedy, Chicago, IL, USA). PD, CAL, and BOP were measured at six sites per tooth for all teeth, excluding third molars. PD was measured as the distance between the gingival margin and the clinical pocket base, CAL was recorded as the distance between the cemento–enamel junction and the clinical pocket base, with both values expressed in millimetres, and the BOP percentage was calculated by dividing the number of sites with BOP by the number of sites explored and multiplying this value by 100. We assessed the Silness and Löe simplified Plaque Index and scored it in six representative Ramfjörd teeth: upper right first molar, upper left central incisor, upper left first premolar, lower left first molar, lower right central incisor, and lower right first premolar. Participants were classified according to three periodontitis stages according to the 2017 World Workshop definition [23]. Subjects were classified as having stage II when interdental CAL at site of greatest loss was 3 to 4 mm and maximum PD ≤ 5 mm, stage III when CAL ≥ 5 mm, PD ≥ 6 mm and tooth loss due to periodontitis of ≤4 teeth, and stage IV when CAL ≥ 5 mm, PD ≥ 6 mm and tooth loss due to periodontitis of ≥5 teeth.

### 2.3. Biochemical Determinations

Venous blood and urine samples were analysed at the Analclinic Laboratory (Mislata-Valencia, Spain). Serum samples were obtained to measure hsCRP and fibrinogen levels as inflammatory markers and acute phase reactants, and malondialdehyde (MDA) levels as oxidative stress markers. Serum hsCRP levels (mg/dL) were quantified using an immunonephelometric assay (Dade Behring, Marburg, Germany), fibrinogen levels (mg/dL) were obtained through the Coagulometry-Thrombin-Clauss time technique using a Solea 100 automatic analyser (Biolabo diagnostics, Maizy, France), and MDA levels (µmol/L) were obtained by the Asakawa-Matsushita method by reacting MDA with 2-thiobarbituric acid, giving a coloured MDA-TBA complex that is quantified using a Uvikon-810 spectrophotometer (Kontron Instruments, Augsburg, Germany). 8-OHdG levels (µg/g creatinine) were tested from urine samples using high-performance liquid chromatography (Analytical HPLC 1200 Series, Agilent Technologies, Santa Clara, CA, USA), which is also an oxidative stress marker.

### 2.4. Statistical Analysis

In reference to sample size determination, the present study was designed in a finite population. Thus, in our case, taking into account that the flow of patients during the selection period can be estimated at 90 periodontal patients/year in the clinic, as well as considering a power of 95% in order to be able to detect differences of ≥0.25 mg/dL between the groups in relation to the primary efficacy criterion (serum hsCRP levels variation), assuming a common standard deviation of 0.20 mg/dL and with an α risk of 0.05. Under these premises, at least 69 subjects were required.

Statistical analyses were performed using SPSS software (IBM Co., Armonk, NY, USA). Continuous variables were expressed as mean and standard deviation for parametric data, qualitative data were expressed as percentages, and proportions were compared using a chi-square test. Continuous variables were compared among groups according to periodontitis stages using a one-way analysis of variance or Kruskal–Wallis test, followed in each case by a post-hoc test. Spearman’s correlation coefficient was used to evaluate the strength of the association between periodontal, inflammatory, and oxidative stress variables. A multivariable regression model was used to evaluate the relationship between two or more explanatory variables, considered as independent variables, and a response variable, considered as a dependent variable, using a stepwise method. A confidence interval of 95% was determined for all tests and a *p*-value <0.05 was considered statistically significant.

## 3. Results

This cross-sectional study analysed 70 subjects with periodontitis. Of these subjects, 27 were men, and 43 were women. Participants were classified according to three periodontitis stages; 22 subjects were classified as having stage II, 30 as having stage III, and 18 as having stage IV, according to the 2017 World Workshop definition [23].

Participant demographic and periodontal parameters are presented in Table 1. No significant differences were found between groups regarding sex. Mean study population age was 53.7 ± 9.3 years, and although the group with advanced periodontitis had a slightly older age, no significant differences were observed between groups regarding age (*p* = 0.055). Most patients in this study did not smoke (65.7%), and no differences were observed in the smoking rate among the three groups as assessed by a chi-square test (*p* = 0.134). Regarding periodontal clinical parameters, PD and CAL progressively worsened with periodontitis severity. Moreover, BOP and the number and percentage of sites with periodontal pockets were higher in patients with stage III and IV periodontal disease than in patients with stage II, whereas the percentage of healthy periodontal sites (PD 1–3 mm) was higher in patients with stage II periodontitis than in patients with stage III and IV. The plaque index was similar between groups (*p* = 0.554).

**Figure 1:**
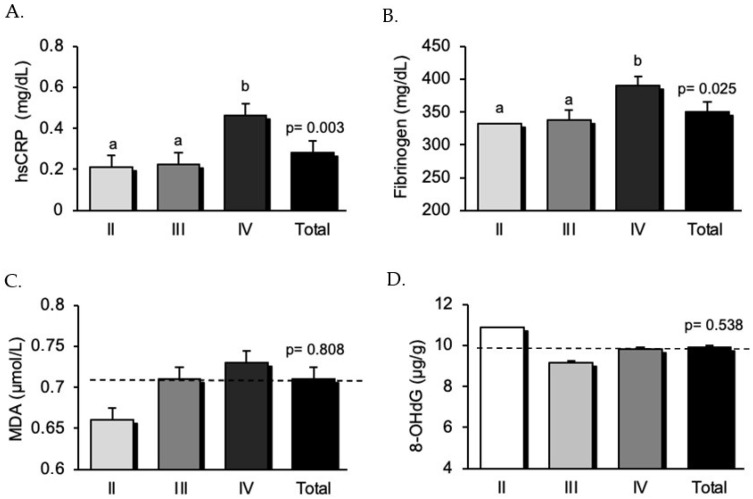
Study population inflammatory and oxidative stress parameters according to periodontitis stages. Serum high-sensitivity C-reactive protein (**A**), fibrinogen (**B**) and malondialdehyde (**C**), and urine 8-hydroxy-2′-deoxyguanosine (**D**) levels. Notes: hsCRP, high-sensitive C-reactive protein; MDA, malondialdehyde; and 8-OHdG, 8-hydroxy-2′-deoxyguanosine. Data are presented as mean ± standard error. Values with different superscript letters (a and b) were significantly different (*p* < 0.05) when the data of patients with different periodontitis stages were compared using a one-way analysis of variance followed by a Student–Newman–Keuls post-hoc test. Hence, means with the same superscript letters are not significantly different (*p* > 0.05), while means with no superscript letters in common are significantly different (*p* < 0.05).

To investigate whether proinflammatory and prooxidative parameters worsen with periodontitis severity, we compared these variables among the three study groups according to periodontitis stage (Figure 1). Systemic inflammatory markers, hsCRP, and fibrinogen, considered as acute phase reactants, were significantly different between groups (*p* = 0.003 and *p* = 0.025, respectively), with higher values in the advanced periodontitis group (stage IV) (Figure 1A,B) than in the other two groups. Mean hsCRP in stage IV 0.463 ± 0.390 mg/dL vs. 0.224 ± 0.203 mg/dL in stage III and 0.210 ± 0.145 mg/dL in stage II. Likewise, mean fibrinogen levels in stage IV 390.6 ± 68.8 mg/dL vs. 338.5 ± 76.5 mg/dL in stage III and 332.2 ± 67.7 mg/dL in stage II. In addition, we determined prooxidative parameter levels, including serum MDA levels and urine 8-OHdG levels. We observed a slight and progressive yet insignificant increase in MDA levels as periodontitis severity increased (0.66 ± 0.18 µmol/L in stage II; 0.71 ± 0.2 µmol/L in stage III; and 0.73 ± 0.26 µmol/L in stage IV. Figure 1C). Likewise, no differences were observed in 8-OHdG urine levels between groups (10.9 ± 3.05 µg/g in stage II; 9.17 ± 2.05 µg/g in stage III; and 9.84 ± 3.21 µg/g in stage IV. Figure 1D).

Correlation coefficients between periodontal, inflammatory, and pro-oxidant parameters from all population are shown in Table 2. PD and CAL, which are periodontal clinical parameters that indicate disease and periodontitis severity, were positively correlated with hsCRP (r = 0.216, *p* = 0.037; and r = 0.234, *p* = 0.026, respectively) and fibrinogen levels (r = 0.320, *p* = 0.007; and r = 0.335, *p* = 0.002, respectively), suggesting a major inflammatory periodontitis component. Moreover, fibrinogen was positively correlated with BOP and the percentage of sites with PD ≥ 6 mm and negatively correlated with the percentage of sites with PD 1–3 mm. In addition, there was a correlation between both proinflammatory parameters, hsCRP, and fibrinogen. Regarding oxidative stress parameters, no correlations were observed between clinical periodontal parameters and serum MDA and urine 8-OHdG levels. However, when we studied bivariate correlations segmenting participants by periodontitis stages, we observed a statistically significant positive correlation between MDA and BOP in patients with stage II periodontitis (r = 0.707, and *p* = 0.025). Finally, we detected bivariate correlations between all analysed periodontal parameters except the plaque index, which was not significantly correlated with CAL and the percentage of sites with PD ≥ 6 mm (data not shown).

As fibrinogen showed significant correlations with several periodontal parameters, we aimed to analyse these associations using a multivariate linear regression analysis. In the multivariable regression model, the association between fibrinogen and correlated variables was evaluated as a potentially independent predictor using the stepwise method. Results showed that hsCRP (β = 0.441) and CAL (β = 0.280) were independently associated with fibrinogen serum levels; this explained 30% of the dependent variable (Table 3).

## 4. Discussion

This study has demonstrated that serum hsCRP and fibrinogen levels are associated with periodontal disease and both parameters increase with periodontitis severity. In this study, we observed a slight and progressive yet insignificant increase in MDA levels as periodontitis severity increased. Likewise, we did not find associations between periodontal parameters and systemic MDA and 8-OHdG levels.

Previous studies suggested a multidirectional association between periodontitis and inflammatory systemic diseases [24]; thus, patients with periodontitis are at greater risk of developing and/or exacerbating diabetes, chronic obstructive pulmonary disease, and cardiovascular diseases, among other conditions [25]. However, these associations remain controversial.

Several epidemiological studies have reported that periodontitis is related to arteriosclerotic events, and this association has been suggested due to the increase in serum levels of proinflammatory and pro-oxidant markers in both diseases, such as TNFα, IL-1, IL-6, and CRP [26,27,28,29], fibrinogen [30], MDA [18,25], and 8-OHdG [13,31]. Recently, a study has even shown that periodontitis-especially severe-is independently associated with a considerable increase in platelet count due to an increase in the systemic inflammation, which could be a new potential link with cardiovascular disease [32]. On the contrary, some studies suggest that this association is mainly due to the existence of common risk factors [33,34]. Some studies only observed an increase in hsCRP levels in some individuals with periodontitis [33], and in other studies, it has been observed that patients with periodontitis showed similar levels of hsCRP in serum compared to edentulous individuals [34]. However, a large number of previous epidemiological studies have shown an association between periodontal and proinflammatory parameters [26,27,28,29,30,35,36], suggesting the possible systemic repercussions that local chronic inflammation could increase the systemic inflammatory burden. In addition, previous studies have shown that non-surgical periodontal therapy was effective in reducing the plasma levels of hsCRP and fibrinogen [37,38]. In line with these findings, in this study, we observed that both serum hsCRP and fibrinogen increased with periodontitis severity. Furthermore, we observed positive associations between periodontal clinical parameters and both inflammatory parameters, especially fibrinogen. When we applied the stepwise method with fibrinogen as the dependent variable and all variables correlated with fibrinogen as independent variables, we observed a significant relationship between hsCRP and CAL, showing the interconnection between different systemic inflammation markers and probing dependent variables with a cumulative profile. Therefore, we suggest that determining serum hsCRP and fibrinogen levels in patients with periodontitis, especially in those with stage III or IV of periodontitis, can be very useful for predicting cardiovascular results. hsCRP is very useful as it represents a global measure of endothelial function [39], and fibrinogen strongly affects blood coagulation, blood rheology, and platelet aggregation and directly affects the vascular wall. All these phenomena might constitute the pathophysiological mechanisms involved in the association between these prominent acute-phase reactants and cardiovascular events [40]. Thus, a possible mechanism potentially linking periodontitis to atherosclerosis is the dumping of inflammatory mediators originating from periodontal lesions into systemic circulation. Such inflammatory mediators, including CRP, matrix metalloproteinases, fibrinogen, and other haemostatic factors, may further accelerate atheroma formation and progression mainly through oxidative stress and inflammatory dysfunction [41].

Most previous periodontal studies evaluated oxidative stress through the global burden of ROS and guanine-derived biomarkers in saliva and gingival crevicular fluid [42,43,44]. However, in this current study, we evaluated fluctuations in both markers in blood and urine; these fluctuations are more commonly related to systemic oxidative damage markers associated with periodontal diseases, such as MDA and 8-OHdG [13,18,19,45,46,47,48].

In a recent meta-analysis, most studies on periodontitis and oxidative stress found a significant increase in MDA levels in the saliva and gingival crevicular fluid of patients with periodontitis compared to non-periodontitis patients [14]. However, in this systematic review, no data on serum MDA levels were collected. In this study, we evaluated MDA levels in serum samples. Although we did not observe statistically significant differences in serum MDA levels among groups with different periodontitis stages, a slight increase in MDA levels can be seen as periodontitis severity increases. Nonetheless, we did not observe significant correlations between periodontal parameters and serum MDA levels. Similar to our study, a previous study did not observe an association between periodontitis and serum MDA levels, although it showed higher saliva MDA levels in patients with periodontitis and a significant correlation between these saliva MDA levels and periodontal parameters [49]. The lack of an association between serum MDA levels and periodontal parameters in this study may be because this oxidative stress parameter was evaluated in a small representative study population sample. Nevertheless, it is worthy to note that a surprising association was found between MDA and BOP when a correlation analysis segmenting the population by stages of periodontitis was performed. Similar to this result, BOP showed a moderate positive correlation with serum MDA levels in a previous study [50]. Gingival bleeding on probing is a sign of local inflammation and periodontal destruction, which can increase proinflammatory marker and oxidative damage systemic levels.

The most frequent oxidative DNA modification usually occurs at guanosine levels, producing 8-OHdG. 8-OHdG levels were used as an index of oxidative DNA damage. Recently, 8-OHdG has been widely used in many studies not only as a biomarker for measuring endogenous oxidative DNA damage, but also as a risk factor for many diseases [48].

Previous studies have detected higher salivary 8-OHdG levels in periodontitis patients than in healthy controls [51] and also significant correlations between salivary 8-OHdG levels and periodontal parameters [20]. In other studies, higher serum 8-OHdG levels have been observed in patients with periodontitis and hyperlipidemia [52], or periodontitis and polycystic ovary syndrome [19] than in patients without periodontal disease associated with systemic pathology. In addition, a recent systematic review with meta-analysis identified increased levels of 8-OHdG in gingival crevicular fluid of periodontitis sites [53]. However, unlike this study, previous studies have not evaluated the urine 8-OHdG levels in patients with periodontitis. We did not observe significant differences in urine 8-OHdG levels between patients with different periodontitis stages. Likewise, we did not observe a significant correlation between urine 8-OHdG levels and periodontal clinical parameters. After DNA repair, 8-OHdG is excreted in the urine, and numerous studies have indicated that urinary 8-OHdG is an important cellular oxidative stress biomarker [48]. Therefore, more studies on patients with periodontitis are required to evaluate systemic blood or urine oxidative stress markers to determine the impact of periodontal disease on the pathophysiology of systemic inflammatory diseases.

To the best of our knowledge, this is the first periodontal study to measure urine 8-OHdG levels to determine the effect of periodontitis on systemic pro-oxidant marker levels. We performed a full-mouth periodontal examination, measuring six sites per tooth for all teeth, thus obtaining more comprehensive data than studies using a partial mouth assessment. However, this study has some limitations. The sample size was small. Therefore, when dividing the sample by periodontitis stages, the groups could individually lose statistical power. MDA and 8-OHdG levels were evaluated only in serum and urine samples, respectively. The grade criterion was not used to classify the groups due to the small sample size and the cross-sectional nature of the study. Finally, the cross-sectional nature of this study limits its interpretability. Undoubtedly, there is still much to learn about the association between systemic proinflammatory and pro-oxidant markers and periodontitis and their relationship with systemic inflammatory disease risk. Further prospective studies should be conducted to further reveal the role of periodontitis on systemic levels of inflammatory and pro-oxidant markers and their respective consequences on general health.

## 5. Conclusions

The findings of this study support the concept that periodontitis, especially advanced periodontitis, increases systemic inflammation mediators’ levels, which are atherosclerotic disease risk factors. We observed an increase in serum of hsCRP and fibrinogen levels as the severity of periodontitis increases. However, we did not find associations between periodontitis severity and MDA serum levels and 8-OHdG urine levels. This suggests the importance of early periodontal diagnosis and treatment to avoid possible future systemic complications due to the persistence of chronic inflammation. Periodontitis, as a local inflammatory disease, could cause an increase in inflammation and consequently in systemic oxidative stress and could be involved in the development or exacerbation of systemic inflammatory diseases, such as cardiovascular disease. Therefore, further prospective studies are needed to evaluate the systemic oxidative stress marker levels in patients with periodontitis to establish possible implications of periodontal disease in systemic inflammatory diseases.

## Figures and Tables

**Table 1:** Descriptive study population parameters according to periodontitis stages.

	Periodontitis			
	Stage II	Stage III	Stage IV	All
Demographic parameters				
n (% females)	22 (21.4)	30 (24.3)	18 (15.7)	70 (61.4)
Age (years)	52.5 ± 10.4	51.9 ± 9.0	58.2 ± 7.2	53.7 ± 9.3
Smoking habit				
Non-smokers % (n)	20.0 (14)	28.6 (20)	17.1 (12)	65.7 (46)
Smokers ≤10 cigarettes/day	10.0 (7)	4.3 (3)	2.9 (2)	17.1 (12)
Smokers >10 cigarettes/day	1.4 (1)	10.0 (7)	5.7 (4)	17.1 (12)
Periodontal parameters				
PD (mm)	2.64 ± 0.35 ^a^	3.51 ± 0.68 ^b^	3.92 ± 0.65 ^c^	3.34 ± 0.77 ***
CAL (mm)	2.80 ± 0.35 ^a^	3.68 ± 0.65 ^b^	4.16 ± 0.68 ^c^	3.53 ± 0.78 ***
Sites PD ≥ 4 mm (n)	27.6 ± 18.8 ^a^	68.6 ± 30.6 ^b^	64.0 ± 23.6 ^b^	54.5 ± 31.3 ***
Sites PD 1–3 mm (%)	82.7 ± 11.6 ^a^	55.7 ± 19.2 ^b^	47.6 ± 17.5 ^b^	62.1 ± 21.9 ***
Sites PD 4–5 mm (%)	16.6 ± 11.8 ^a^	31.7 ± 12.2 ^b^	36.9 ± 11.6 ^b^	28.3 ± 14.3 ***
Sites PD ≥ 6 mm (%)	1.07 ± 0.85 ^a^	12.3 ± 10.6 ^b^	15.6 ± 9.4 ^b^	9.61 ± 10.2 ***
BOP (%)	21.4 ± 17.2 ^a^	32.4 ± 21.3 ^a,b^	44.4 ± 32.4 ^b^	32.0 ± 24.8 **
Plaque index (A.U)	0.71 ± 0.75	0.72 ± 0.63	0.93 ± 0.91	0.77 ± 0.74

Notes: PD, probing depth; CAL, clinical attachment loss; BOP, bleeding on probing; and A.U, arbitrary units. Data are presented as mean ± standard deviation or percentages (n). ** *p* < 0.01; and *** *p* < 0.001 when patients with different periodontitis stages were compared with an analysis of variance test. Values with different superscript letters (^a, b^ and ^c^) were significantly different when the three groups were compared using a Student–Newman–Keuls post-hoc test. Hence, means with the same superscript letters are not significantly different (*p* > 0.05), while means that have no superscript letters in common are significantly different (*p* < 0.05).

**Table 2:** Spearman’s correlation coefficients between periodontal, inflammatory, and oxidative stress parameters.

	hsCRP		Fibrinogen		MDA		8-OHdG	
	r	*p*	r	*p*	r	*p*	r	*p*
PD	**0.216**	**0.037**	**0.320**	**0.007**	−0.245	0.183	−0.030	0.876
CAL	**0.234**	**0.026**	**0.335**	**0.005**	−0.229	0.214	−0.041	0.831
n Sites PD ≥ 4 mm	0.044	0.717	0.226	0.062	−0.142	0.445	−0.061	0.751
% Sites PD 1–3 mm	−0.119	0.330	**−0.264**	**0.028**	0.190	0.305	0.076	0.695
% Sites PD 4–5 mm	0.113	0.353	0.231	0.056	0.009	0.960	0.025	0.898
% Sites PD ≥ 6 mm	0.188	0.121	**0.263**	**0.029**	−0.186	0.315	−0.032	0.869
% BoP	0.137	0.261	**0.311**	**0.009**	0.062	0.740	0.046	0.814
Plaque index	0.043	0.726	−0.057	0.644	0.237	0.199	−0.048	0.806
hsCRP	----	----	**0.490**	**<0.001**	0.191	0.302	−0.137	0.478
Fibrinogen	**0.490**	**<0.001**	----	----	−0.098	0.605	−0.204	0.290
MDA	0.191	0.302	−0.098	0.605	----	----	0.244	0.202
8-OHdG	−0.137	0.978	−0.204	0.290	0.244	0.202	----	----

Notes: PD, probing depth; CAL, clinical attachment loss; BOP, bleeding of probing; hsCRP, high-sensitivity C-reactive protein; MDA, malondialdehyde; and 8-OHdG, 8-hydroxy-2′-deoxyguanosine. Values in bold represent statistically significant correlations (*p* < 0.05).

**Table 3:** Stepwise multivariable regression model with fibrinogen as a dependent variable.

Dependent Variable	Independent Variables	Unstandardized Coefficients		Standardized Coefficients	*p*-Value
		B	SE	β	
Fibrinogen	hsCRP	11.96	2.83	0.441	<0.001
	CAL	26.14	9.74	0.280	0.009
	Multiple R square adjusted			0.304	
		R		0.570	
		*p*		<0.001	

PD, BOP, percentage of sites with PD 1–3 mm, and PD ≥ 6 mm were excluded from the model because they were not significant predictors (*p* > 0.05). Notes: hsCRP, high-sensitivity C-reactive protein; CAL, clinical attachment loss; PD, probing depth; and BOP, bleeding on probing.

## Data Availability

These data will be treated in accordance with the provisions of Regulation (EU) 2016/679 of April 27 (GDPR) and Organic Law 3/2018 of April 5 December (LOPDGDD), for which the following treatment information is provided. Purposes of the treatment: -Participation of the interested party in the Study on the relationship between periodontal disease and serum levels of ultrasensitive hsCRP and Fibrinogen. -Statistical and/or scientific purposes. Data conservation criteria: they will be kept for no longer than necessary to maintain the end of the treatment and when it is no longer necessary for that purpose, they will be deleted with adequate security measures to guarantee the pseudonymization of the data or the total destruction of the same. Communication of the data: the data will not be communicated to third parties, except legal obligation. Rights that assist the Interested Party: -Right to withdraw consent at any time. -Right of access, rectification, portability and deletion of your data and the limitation or opposition to its treatment. -Right to file a claim with the Control Authority (www.aepd.es) if you consider that the treatment does not comply with current regulations.
